# Efficacy and safety of intermittent intravenous doxercalciferol in the treatment of secondary hyperparathyroidism in Chinese patients on maintenance hemodialysis: a phase II, open-label, prospective, multicenter study

**DOI:** 10.3389/fphar.2025.1574679

**Published:** 2025-04-11

**Authors:** Guang Yang, Yaoyu Huang, Yifei Ge, Xiangbao Yu, Li Liu, Li Xiang, Bin Liu, Chaoqing Gao, Changhua Liu, Yong Xu, Wenwen Li, Donghua Lou, Tongqiang Liu, Huijuan Mao

**Affiliations:** ^1^ Department of Nephrology, The First Affiliated Hospital of Nanjing Medical University, Nanjing, China; ^2^ Department of Nephrology, The Second People’s Hospital of Hefei, Hefei, China; ^3^ Department of Nephrology, The First People’s Hospital of Changzhou, Changzhou, China; ^4^ Department of Nephrology, The People’s Hospital of Wuxi, Wuxi, China; ^5^ Department of Nephrology, Yijishan Hospital, The First Affiliated Hospital of Wannan Medical College, Wuhu, China; ^6^ Department of Nephrology, Northern Jiangsu People’s Hospital, Yangzhou, China; ^7^ Department of Nephrology, The Second People’s Hospital of Huai’an, Huai’an, China; ^8^ Department of Nephrology, Sir Run Run Hospital, Nanjing Medical University, Nanjing, China; ^9^ Nanjing Yike Baoda Pharmaceutical Technology Co., Ltd., Nanjing, China; ^10^ Department of Nephrology, The Second People’s Hospital of Changzhou, The Third Affiliated Hospital of Nanjing Medical University, Changzhou, Jiangsu, China

**Keywords:** secondary hyperparathyroidism, doxercalciferol, maintenance hemodialysis, efficacy, safety

## Abstract

**Objective:**

This phase II, open-label, prospective, multicenter study evaluated the efficacy and safety of intermittent intravenous doxercalciferol in treating secondary hyperparathyroidism (SHPT) in Chinese maintenance hemodialysis (MHD) patients.

**Methods:**

MHD patients aged 18 to 75 years with a serum intact parathyroid hormone (iPTH) level of 400 pg/mL or higher were enrolled and stratified into Mild, Moderate, and Severe groups based on baseline iPTH levels (400–599.99, 600–799.99, and ≥800 pg/mL, respectively). Patients received an initial dose of 4 μg of doxercalciferol three times weekly for 12 weeks, with subsequent dose adjustments to target iPTH levels of 150–300 pg/mL.

**Results:**

Of the 45 patients enrolled, 44 completed the study, with 20 patients in the Mild Group, 12 in the Moderate Group, and 12 in the Severe Group. The baseline iPTH level for the 44 patients was 655.05 (469.68, 831.40) pg/mL, which decreased to 269.90 (176.45, 365.65) pg/mL after 12 weeks of treatment. The overall mean percentage change in iPTH levels from baseline to week 12 was −55.45% ± 20.08%, with 86.4% of patients (38 cases) achieving a ≥30% reduction compared to baseline. At week 12, 80.00% of patients (16 cases) in the Mild Group had iPTH levels within the target range of 150–300 pg/mL or less than 150 pg/mL, compared to 41.76% (5 cases) in the Moderate Group and 33.33% (4 cases) in the Severe Group. All three groups showed a decrease in serum alkaline phosphatase (ALP) levels, with the Severe Group experiencing a statistically significant reduction (P = 0.001). The most common adverse event was hypercalcemia, occurring in 33.3% of patients (15 cases), with only 8.9% (4 cases) experiencing severe hypercalcemia (serum calcium >2.8 mmol/L). Hypercalcemia was resolved after dose reduction or discontinuation of the medication.

**Conclusion:**

Intermittent intravenous doxercalciferol effectively reduces iPTH levels in Chinese MHD patients, with a manageable safety profile. While hypercalcemia is a concern, the incidence of severe cases is not high. This study supports doxercalciferol as a potential treatment option for SHPT in Chinese MHD patients.

**Clinical Trial Registration:** https://www.chictr.org.cn/showproj.html?proj=187332, identifier ChiCTR2300073196.

## Introduction

Secondary hyperparathyroidism (SHPT) is a prevalent and severe complication in patients with chronic kidney disease (CKD), particularly among those undergoing maintenance hemodialysis (MHD). SHPT is characterized by elevated levels of parathyroid hormone (PTH), leading to disturbances in calcium and phosphorus homeostasis, bone disorders, and increased cardiovascular risks. The pathogenesis of SHPT involves a complex interplay of factors, including hypocalcemia, hyperphosphatemia, and decreased production of active vitamin D (1,25-dihydroxyvitamin D3) (Zaimi and Graspa, 2024). As the global prevalence of end-stage renal disease (ESRD) continues to rise, effective management of SHPT has become a critical aspect of CKD care.

Current treatment strategies for SHPT include dietary phosphorus restriction, phosphate binders, active vitamin D analogs (VDRA), and calcimimetics. Among these, active vitamin D analogs have been widely used due to their ability to suppress PTH secretion and improve calcium and phosphorus balance. However, the use of traditional vitamin D analogs is often limited by their potential to induce hypercalcemia and hyperphosphatemia, which can exacerbate cardiovascular risks and vascular calcification. Doxercalciferol, a synthetic vitamin D2 analog, is derived from ergocalciferol (vitamin D2), which is synthesized by plants when they are exposed to ultraviolet (UV) light. When ergocalciferol is subjected to a series of chemical modifications, it can be converted into doxercalciferol (Holick, 2007). Doxercalciferol was developed by Genzyme in the United States and was approved by the U.S. FDA for the treatment of SHPT in 2000. Ducalcitriol is a selective VDRA that mainly acts on the parathyroid gland, reducing parathyroid hormone levels by inhibiting the synthesis and secretion of parathyroid hormone, with minimal impact on bones and intestines, and increasing blood calcium and phosphorus concentrations to a lesser extent (Maung, et al., 2001).

An epidemiological survey indicates that the prevalence of CKD in China is approximately 8.2% (Wang et al., 2023). As chronic kidney disease progresses, the incidence of SHPT gradually increases. Given the notably high prevalence of CKD and SHPT in China, where SHPT due to CKD is the most prevalent globally at 85.14%, there is an urgent need for effective and safe treatment options (Wang et al., 2024). The lack of approval for doxercalciferol in China highlights a gap in the available therapeutic arsenal for managing SHPT in this population. There is a paucity of data regarding the use of intermittent intravenous dosing of the vitamin D sterols doxercalciferol in the Chinese population, particularly in the context of MHD. This open-label, prospective, stratified, multicenter study aims to evaluate the efficacy and safety of intermittent intravenous doxercalciferol in the treatment of SHPT in Chinese patients on MHD.

## Materials and methods

### Patients

This study is designed as a Phase II, open-label, prospective, stratified, multicenter clinical study to evaluate the efficacy and safety of intermittent intravenous (IV) doxercalciferol in the treatment of secondary hyperparathyroidism (SHPT) in Chinese patients undergoing maintenance hemodialysis (MHD). Chinese Clinical Trials Registry Registration number: ChiCTR2300073196 (https://www.chictr.org.cn/showproj.html?proj=187332). The study will be conducted across multiple dialysis centers in Jiangsu and Anhui provinces of China to ensure a diverse patient population and generalizability of the results. Patients were recruited from participating dialysis centers.

Stratification Criteria: Participants will be stratified into three groups based on their baseline intact parathyroid hormone (iPTH) levels (Qiu et al., 2017; Pereira, et al., 2023).

1) Mild Group: iPTH 400–599.99 pg/mL
2) Moderate Group: iPTH 600–799.99 pg/mL
3) Severe Group: iPTH≥800 pg/mL

Stratification by iPTH levels aims to ensure balanced representation across different severities of SHPT and to assess the efficacy and safety of doxercalciferol in each subgroup.

Inclusion Criteria: To be eligible for enrollment, patients must meet the following criteria.

1) Age: 18–75 years old (as calculated from the date of signing the informed consent form), either gender.
2) Chronic Kidney Disease (CKD): Patients with CKD undergoing maintenance hemodialysis for at least 3 months with a stable dialysis regimen (dialysis frequency of 3 times per week, dialysis duration of 4 h ± 0.25 h per session, dialysis fluid calcium concentration of 1.5 mmol/L). The dialysis regimen is not expected to undergo significant changes during the study period.
3) Intact Parathyroid Hormone (iPTH) Level: Serum iPTH≥400 pg/mL at the end of the washout period.
4) Serum Phosphorus Level: Average serum phosphorus level during the washout period of 0.8–2.2 mmol/L (2.5–6.9 mg/dL, inclusive of boundary values).
5) Corrected Serum Calcium Level: Average corrected serum calcium level during the washout period ≤ 2.6 mmol/L (10.5 mg/dL).
6) Contraception: Subjects agree to use effective contraceptive measures during the trial and for 3 months after the trial to avoid pregnancy for themselves and their partners.
7) Communication and Compliance: Subjects are able to maintain good communication with the investigator, fully understand the purpose, process, and risks of the trial, agree to comply with all requirements of the clinical trial, and voluntarily sign the informed consent form.

Exclusion Criteria: Patients will be excluded from the study if they meet any of the following criteria.

1) Allergic Conditions: Individuals prone to allergic reactions such as rash or urticaria; those allergic to the active ingredient of the study drug or any excipients in the formulation; individuals with a history of severe drug allergies (e.g., anaphylactic shock).
2) Recent Treatment with Specific Medications: Treatment with bisphosphonates, denosumab, or teriparatide within 24 weeks prior to screening.
3) Recent Use of Vitamin D and Related Therapies: Receipt of vitamin D, its analogs, or calcimimetic agents during the washout period.
4) Life Expectancy: Patients with a life expectancy of less than 1 year.
5) Uncontrolled Diabetes: A history of poorly controlled diabetes (defined as glycated hemoglobin above 9.5% despite long-term hypoglycemic therapy).
6) Refractory Hypertension: A history of refractory hypertension (defined as systolic blood pressure above 180 mmHg or diastolic blood pressure above 110 mmHg despite antihypertensive medication).
7) Severe Cardiac Conditions: Patients with severe cardiac diseases, such as acute myocardial infarction, cardiogenic shock, acute heart failure, or chronic congestive heart failure (NYHA class ≥ III), or a history of severe cardiac arrhythmias.
8) Severe Pulmonary Insufficiency: Patients with severe pulmonary insufficiency.
9) Severe Liver Disease: Patients with severe liver disease (ALT, AST, TBIL > 2 times the upper limit of normal).
10) Infectious Disease Markers: Individuals with positive results for human immunodeficiency virus antibody (HIV-Ab), hepatitis B virus surface antigen (HBsAg), hepatitis C virus antibody (HCV-Ab), or syphilis antibody (TP-Ab) within the past 6 months.
11) Recent Acute Illnesses: Patients who have experienced acute infectious diseases, acute brain diseases, or acute gastrointestinal bleeding within the past 3 months.
12) Parathyroid Interventions: Patients who have undergone or are planning to undergo parathyroid intervention, parathyroidectomy, or kidney transplantation during the trial period.
13) Recent Participation in Other Clinical Trials: Participation in other clinical trials and receipt of investigational drugs or device interventions within 3 months prior to screening.
14) Pregnancy and Contraception: Women who are currently breastfeeding, pregnant, or unable to use effective contraceptive measures during the trial.
15) Other Ineligibility: Any other condition that, in the opinion of the investigator, makes the subject unsuitable for participation in the clinical study.

The institutional review board at each institution approved the protocols and consent forms for the trial; informed consents for the protocol were obtained from each patient.

### Drug

The doxercalciferol injection in this clinical study is a Category three chemical drug developed by Nanjing Heron Pharmaceutical Science and Technology Co., Ltd. This product has the same active ingredient, dosage form, strength, indications, route of administration, and dosage regimen as the reference listed drug. The formulation process of the calcitriol injection was studied with reference to the reference listed drug’s prescribing information and related literature, and the formulation is consistent with that of the reference listed drug. Previously, pharmaceutical and non-clinical studies supporting the application for clinical trials were completed, and the clinical trial application materials for calcitriol injection were submitted to the National Medical Products Administration in June 2022, accepted in July 2022, and the “Drug Clinical Trial Approval Notice” (Approval No.: 2022LP01616) was obtained on 28 September 2022. This product has undergone long-term stability studies, and the results show that the stability trend of this product is basically consistent with that of the reference listed drug, indicating that the quality of this product is consistent with that of the reference listed drug.

### Study design

After the patients sign the informed consent form, they will discontinue the current use of vitamin D and its analogues, as well as calcimimetics. During the washout period, the use of phosphate binders (calcium-based or non-calcium-based) is permitted, and the dose and type can be adjusted as appropriate. After an 8-week washout period, the inclusion and exclusion criteria will be verified, and the study drug, doxercalciferol injection, will be administered. The treatment period will last for 12 weeks, with intravenous bolus administration at the end of each dialysis session. The initial dose will be 4 μg, administered three times a week. The dose will be adjusted based on the results of serum iPTH and corrected serum calcium tests, with a maximum weekly dose of 18 μg.

### Dose adjustments

During the intravenous doxercalciferol trial, the dose of doxercalciferol was adjusted, as described subsequently, to bring plasma iPTH levels into a target range of 150 to 300 pg/mL (National Kidney Foundation, 2003). These values are associated with normal or near-normal bone formation and minimal features of secondary hyperparathyroidism among hemodialysis patients. If at the sixth week of treatment the iPTH level is > 300 pg/mL and has not been suppressed by 50% or more compared to the baseline, the dose will be increased by 1 μg on the next dialysis day after the results are known. To ensure the safety of the subjects, the serum calcium value must be at 2.6 mmol/L (10.5 mg/dL) or below before increasing the dose. If during the treatment period the iPTH value drops to below 150 pg/mL, the drug will be discontinued for 1 week starting on the next dialysis day after the results are known, and then the dose will be reduced by 1 μg to resume administration. If during the treatment period the corrected serum calcium exceeds 2.8 mmol/L (11.2 mg/dL), the drug must be temporarily discontinued until the pre-dialysis corrected serum calcium level drops to 2.6 mmol/L (10.5 mg/dL) or below. After that, the dose can be reduced by 1 μg to resume administration. If during the treatment period the corrected serum calcium is ≥ 2.6 mmol/L (10.5 mg/dL) but ≤2.8 mmol/L (11.2 mg/dL), the dose will be reduced by 1 μg on the next dialysis day after the results are known.

### Laboratory examinations

Laboratory examinations included measurements of serum alkaline phosphatase, albumin, glucose, creatinine, urea, uric acid, intact parathyroid hormone (iPTH), phosphorus, corrected calcium, and glycated hemoglobin. These parameters were assessed at specific time points throughout the study. At weeks 0 (baseline), 6, and 12: Serum alkaline phosphatase, albumin, glucose, creatinine, urea, uric acid, iPTH, phosphorus, corrected calcium, and glycated hemoglobin were measured. At weeks 2, 4, 8, and 10: Serum iPTH, corrected calcium, and phosphorus were measured. All laboratory analyses were performed at a central laboratory to ensure consistency and reliability of the results.

### Statistical analysis

Statistical analysis was performed using SPSS 26.0 statistical software. For measurement data that conformed to a normal distribution, results were expressed as mean ± standard deviation (x ± s). Comparisons among multiple groups were conducted using one-way analysis of variance (ANOVA), and *post hoc* pairwise comparisons were performed using the Least Significant Difference (LSD) test. For multiple groups across multiple time points, a two-way repeated measures ANOVA was employed. Initially, Mauchly’s sphericity test was conducted; if the sphericity assumption was met, the uncorrected results were used. If the sphericity assumption was not met, the Greenhouse-Geisser correction was applied, and *post hoc* pairwise comparisons were conducted using the Bonferroni test. For data that did not conform to a normal distribution, results were expressed as median (interquartile range). Comparisons among multiple groups were performed using the Kruskal-Wallis rank-sum test, and *post hoc* pairwise comparisons were conducted using the Bonferroni test. For the main effect comparison of multiple groups across multiple time points, the generalized estimating equation (GEE) was used. For single time point comparisons among multiple groups, the Kruskal-Wallis rank-sum test was employed, and *post hoc* pairwise comparisons were conducted using the Bonferroni test. For within-group comparisons across multiple time points in a single group, the Friedman test was used, and *post hoc* pairwise comparisons were conducted using the Bonferroni test. Categorical data were expressed as frequency (percentage). Comparisons between groups were performed using the chi-square test or the corrected chi-square test. A P-value less than 0.05 was considered statistically significant.

## Results

### Patient characteristics

Between May 2023 to March 2024, A total of 97 patients were screened for this study, of which 45 were enrolled. One patient withdrew, and the remaining 44 patients from nine dialysis centers in Jiangsu Province and Anhui Province completed the clinical trial. In the 44 patients who completed the clinical trial, there were 34 (77.3%) males and 10 (22.7%) females, with an average age of 50.61 ± 11.02 years. Initial iPTH levels were categorized into three groups: the Mild Group with initial iPTH levels of 400–599.99 pg/mL, comprising 45.45% of the patients (n = 20); the Moderate Group with initial iPTH levels of 600–799.99 pg/mL, representing 27.27% of the patients (n = 12); and the Severe Group with initial iPTH levels of 800 pg/mL and above, accounting for 27.27% of the patients (n = 12) (Table 1).

**TABLE 1:** Summary of baseline demographic and clinical characteristics (n = 44).

Parameter	Total (*n* = 44)	Mild group (*n* = 20)	Moderate group (*n* = 12)	Severe group (*n* = 12)	*χ* ^2^/*F/H*	*P* value
Gender, *n* (%)					1.103	0.576
Male	34 (77.3)	14 (70.0)	10 (83.3)	10 (83.3)		
Female	10 (22.7)	6 (30.0)	2 (16.7)	2 (16.7)		
Age (years)	50.61 ± 11.02	51.65 ± 10.04	50.83 ± 10.36	48.67 ± 13.69	0.268	0.766
Weight (kg)	65.00 (59.73,73.50)	66.10 (54.18,74.63)	64.00 (58.13,72.15)	65.95 (61.05,73.48)	0.273	0.873
BMI (kg/m^2^)	23.12 (21.29,24.64)	23.59 (20.51,24.92)	22.75 (20.91,23.87)	23.22 (22.26,24.67)	0.923	0.630
Alkaline Phosphatase (ALP)	133.44 ± 83.21	105.77 ± 31.66	122.19 ± 46.62	190.81 ± 134.31^a^	4.783	0.014
Albumin (ALB)	41.46 ± 2.58	41.49 ± 3.14	41.13 ± 1.97	41.77 ± 2.24	0.179	0.837
Creatinine (Cr)	1,011.83 ± 183.32	1,026.86 ± 167.88	1,069.29 ± 201.46	929.31 ± 175.10	1.956	0.154
Urea (UREA)	21.08 (18.72,27.11)	20.90 (18.86,26.73)	23.23 (19.46,27.15)	19.82 (15.87,27.11)	1.348	0.510
Serum iPTH	655.05 (469.68,831.40)	463.15 (429.00,528.70)	706.75 (673.48,733.93)^a^	952.15 (870.28,1158.25)^ab^	37.239	<0.001
Serum Phosphorus	1.76 ± 0.31	1.70 ± 0.33	1.75 ± 0.31	1.86 ± 0.27	1.022	0.369
Corrected Serum Calcium	2.29 ± 0.14	2.25 ± 0.15	2.29 ± 0.14	2.34 ± 0.13	1.338	0.274

Note: Compared with the mild group, a P < 0.05; compared with the moderate group, b P < 0.05.

### Control of iPTH

After 12 weeks of treatment, the mean percentage change in iPTH values relative to baseline was statistically significant. For the 44 patients, the percentage change in iPTH values at week 12 compared to baseline was −55.45% ± 20.08%, with a minimum-maximum range of −84.41%–2.67%, P < 0.0001 (Table 2).

**TABLE 2:** Serum iPTH relative to baseline after 12 weeks of treatment in all patients.

Parameter	Baseline	Week 12	Value change from baseline at week 12	Percentage change from baseline at week 12	*t*	*P* value
M(Q1∼Q3)	655.05 (469.68,831.40)	269.90 (176.45,365.65)	/	/	11.06	<0.0001
Min∼Max	409.7–2039	65.62–990.9	−1,127.2–15.1	−84.41%–2.67%	/	/
Mean ± Std	/	/	−395.16 ± 237.02	−55.45% ± 20.08%	18.31	<0.0001

Compared with baseline, the iPTH levels in the Mild, Moderate, and Severe Groups decreased at week 12 of treatment, and the differences were statistically significant (Table 3).

**TABLE 3:** Serum iPTH Relative to Baseline After 12 Weeks of Treatment in three groups.

Group	Baseline	Week 12	*χ* ^2^	*P* value
Mild Group (*n* = 20)	463.15 (429.00,528.70)	178.70 (146.28,282.83)	47.421	<0.001
Moderate Group (*n* = 12)	706.75 (673.48,733.93)^a^	316.40 (231.55,360.93)^a^	37.571	<0.001
Severe Group (*n* = 12)	952.15 (870.28,1158.25)^a,b^	419.20 (221.33,695.38)^a^	33.536	<0.001

Note: Compared with the mild group, a P < 0.05; compared with the moderate group, b P < 0.05.

**FIGURE 1:**
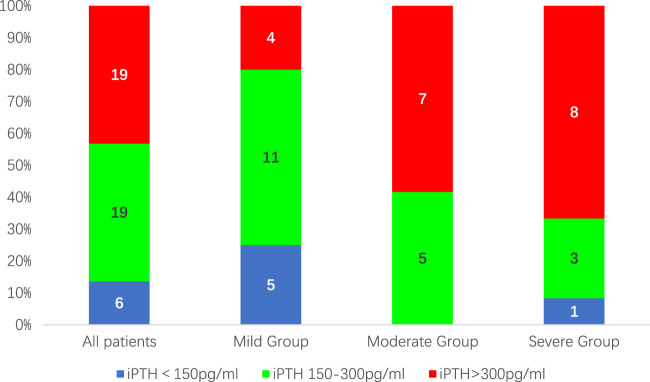
The proportion of patients with iPTH values within the target range of 150–300 pg/mL after 12 weeks of treatment.

**FIGURE 2:**
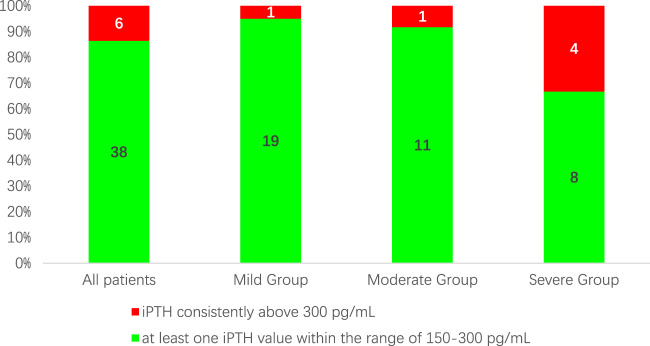
The proportion of patients with at least one iPTH value within the range of 150–300 pg/mL during the 12-week treatment period.

After 12 weeks of treatment, 38 patients (86.4%) had a reduction in iPTH levels of ≥30% compared to baseline. At the same time, the proportion of patients with iPTH values within the K/DOQI clinical practice guidelines target range of 150–300 pg/mL or below 150 pg/mL was 25(56.82%), 16(80.00%), 5(41.76%), and 4 (33.33%) cases for all patients and patients in the mild, moderate, and severe groups, respectively (Figure 1). During the 12-week treatment period, 38 individuals (86.4%) had at least one iPTH value within the range of 150–300 pg/mL (Figure 2).

### Changes in alkaline phosphatase

After 12 weeks of treatment, compared with the baseline, the alkaline phosphatase (ALP) levels in all three groups showed decrease. The decreases in ALP in the mild and moderate groups were not statistically significant (χ2 = 1.728, P = 0.191; χ2 = 2.210, P = 0.123); however, the decrease in ALP in the severe group was statistically significant (χ2 = 8.489, P = 0.001) (Table 4).

**TABLE 4:** Changes in Serum alkaline phosphatase (ALP) Relative to Baseline After 12 Weeks of Treatment in three groups.

Group	Baseline	Week 12	*χ* ^2^	*P* value
Mild Group (*n* = 20)	105.77 ± 31.66	85.44 ± 27.18	1.728	0.191
Moderate Group (*n* = 12)	122.19 ± 46.62	92.27 ± 28.94	2.210	0.123
Severe Group (*n* = 12)	190.81 ± 134.31^b^	132.58 ± 75.83^ab^	8.489	0.001

Note: Compared with Visit 3, a P < 0.05; compared with the mild group, b P < 0.05.

### Therapy duration and dose

The average weekly dose of medication used by all patients was 9.87 ± 2.17 μg, with the lowest weekly dose being 5.3 μg and the highest weekly dose being 13 μg. The differences in medication doses among the three groups were not statistically significant (Table 5).

**TABLE 5:** Comparison of medication doses (μg) at different visit times among the three groups.

Visit Time		Mild group (*n* = 20)		Moderate group (*n* = 12)		Severe group (*n* = 12)		H		P value
baseline		4.00 (4.00,4.00)		4.00 (4.00,4.00)		4.00 (4.00,4.00)		<0.001		1.000	
Week 2		4.00 (4.00,4.00)		4.00 (4.00,4.00)		4.00 (4.00,4.00)		2.040		0.361	
Week 4		3.50 (3.00,4.00)		4.00 (3.25,4.00)		4.00 (4.00,4.00)		5.632		0.060	
Week 6		3.00 (2.25,4.00)		4.00 (3.00,4.00)		4.00 (4.00,4.00)		4.717		0.095	
Week 8		3.00 (2.00,4.00)^a^		4.00 (2.00,4.00)		4.00 (3.25,4.00)		3.811		0.149	
Week 10		3.00 (2.00,3.00)^a^		4.00 (2.00,4.75)		4.00 (3.00,4.00)		4.045		0.132	
Week 12		3.00 (2.00,3.50)^a^		4.00 (2.75,5.00)		4.00 (3.00,4.00)		5.875		0.053	

Note: Compared with baseline, a P < 0.05.

### Drug safety profile

Abnormalities in corrected serum calcium and serum phosphorus, along with other adverse reactions, were monitored at weeks 2, 4, 6, 8, 10, and 12 of the treatment period. 17 patients experienced 22 adverse reactions, with an incidence rate of 37.8%. The adverse reactions that occurred were as follows: hypercalcemia [corrected serum calcium >2.6 mmol/L (10.5 mg/dL)] in 15 cases (33.3%, 19 occurrences); The number of cases with serum calcium >2.8 mmol/L was 4 (8.9%). Hyperphosphatemia [serum phosphorus>2.2 mmol/L (6.9 mg/dL)] in 1 case (2.2%, one occurrence); rash in 1 case (2.2%, one occurrence); and elevated aspartate aminotransferase in 1 case (2.2%, one occurrence).

During the treatment process, the differences in corrected serum calcium levels among the three groups were statistically significant (Table 6), while the differences in serum phosphorus levels among the groups were not statistically significant (Table 7). In the mild, moderate, and severe groups, there were 11 (9.17%), 9 (12.50%), and 7 (9.72%) instances of corrected serum calcium levels >2.6 mmol/L, respectively, and 1 (0.83%), 1 (1.39%), and 2 (2.78%) instances of corrected serum calcium levels >2.8 mmol/L, respectively. Hypercalcemia was resolved after dose reduction or discontinuation of the medication.

**TABLE 6:** Corrected serum calcium levels at different visit times among the three groups.

Visit Time		Mild group (*n* = 20)		Moderate group (*n* = 12)		Severe group (*n* = 12)		F		P value
baseline		2.25 ± 0.15		2.29 ± 0.14		2.34 ± 0.13		1.338		0.274	
Week 2		2.37 ± 0.16^a^		2.37 ± 0.14		2.41 ± 0.13		0.309		0.736	
Week 4		2.43 ± 0.15^a^		2.44 ± 0.19^a^		2.46 ± 0.14		0.100		0.905	
Week 6		2.41 ± 0.14^a^		2.43 ± 0.15^a^		2.48 ± 0.15^a^		1.023		0.368	
Week 8		2.40 ± 0.15^a^		2.41 ± 0.14		2.50 ± 0.16^a^		1.916		0.160	
Week 10		2.41 ± 0.16^a^		2.46 ± 0.13^a^		2.43 ± 0.12		0.524		0.596	
Week 12		2.38 ± 0.13^a^		2.43 ± 0.14^a^		2.49 ± 0.09^a^		2.875		0.068	
F		7.814		4.901		4.079		/		/	
P value		<0.001		0.001		0.003		/		/	

Note: Compared with baseline, a P < 0.05.

**TABLE 7:** Serum phosphorus levels at different visit times among the three groups.

Visit Time		Mild group (*n* = 20)		Moderate group (*n* = 12)		Severe group (*n* = 12)		F		P value
baseline		1.70 ± 0.33		1.75 ± 0.31		1.86 ± 0.27		1.022		0.369	
Week 2		1.80 ± 0.39		1.70 ± 0.30		1.86 ± 0.26		0.714		0.496	
Week 4		1.71 ± 0.34		1.80 ± 0.31		1.72 ± 0.27		0.328		0.722	
Week 6		1.78 ± 0.46		1.59 ± 0.21		1.94 ± 0.33		2.515		0.093	
Week 8		1.73 ± 0.41		1.72 ± 0.34		1.82 ± 0.27		0.313		0.733	
Week 10		1.74 ± 0.43		1.68 ± 0.32		1.80 ± 0.42		0.247		0.782	
Week 12		1.77 ± 0.39		1.83 ± 0.47		1.92 ± 0.40		0.469		0.629	
F		0.494		1.790		1.344		/		/	
P value		0.808		0.129		0.264		/		/	

## Discussion

The results of this open-label, prospective, multicenter study provide valuable insights into the efficacy and safety of intermittent intravenous doxercalciferol in the management of secondary hyperparathyroidism (SHPT) in Chinese patients undergoing maintenance hemodialysis (MHD). The study’s findings are particularly relevant given the high prevalence of chronic kidney disease (CKD) and SHPT in China (Wang et al., 2023), where effective and safe treatment options are urgently needed.

### Efficacy of doxercalciferol in controlling iPTH levels

The primary objective of this study was to evaluate the efficacy of intermittent intravenous doxercalciferol in reducing parathyroid hormone (iPTH) levels. The results demonstrated a statistically significant reduction in iPTH levels after 12 weeks of treatment. The mean percentage change in iPTH values from baseline was −55.45% ± 20.08%, with a significant proportion of patients (86.4%) achieving a reduction of ≥30% compared to baseline. This is clinically meaningful, as it indicates a substantial suppression of PTH secretion, which is crucial for mitigating the adverse effects of SHPT on bone metabolism and cardiovascular health. In comparison, The clinical trial data for doxercalciferol before its approval by the U.S. FDA (Tan, et al., 1997), which employed an open-label, single-arm, before-and-after design with 70 enrolled subjects, showed a baseline iPTH mean of 736 pg/mL. After 12 weeks of treatment, the mean iPTH level decreased to 418 pg/mL, representing a percentage change of −43.21% from baseline. The findings of the current study indicate a slightly greater reduction in iPTH levels compared to U.S. doxercalciferol trial, further supporting the efficacy of doxercalciferol in managing SHPT in Chinese patient population. Several recent studies have corroborated the efficacy of doxercalciferol in reducing iPTH levels in dialysis patients. For instance, in a modified, double-blinded, controlled trial involving 138 hemodialysis patients with secondary hyperparathyroidism, intermittent oral doxercalciferol (1α-hydroxyvitamin D_2_) effectively suppressed iPTH levels with minimal increases in serum calcium and phosphorus, achieving a 70% reduction in iPTH in 80% of patients and reaching target levels in 83%, while maintaining acceptable mild hypercalcemia and hyperphosphatemia during the 16-week open-label and 8-week double-blinded treatment phases (Frazão, et al., 2000). In a study of 60 pediatric peritoneal dialysis patients with secondary hyperparathyroidism, doxercalciferol was as effective as calcitriol in controlling serum parathyroid hormone levels and suppressing bone formation rates, with sevelamer allowing for higher vitamin D doses, while both phosphate binders equally controlled serum phosphate levels, but calcium carbonate increased serum calcium, and FGF-23 levels rose over fourfold with both vitamin D treatments (Wesseling-Perry, et al., 2011). Another retrospective cohort study involving 4,560 hemodialysis patients compared the clinical outcomes of doxercalciferol/paricalcitol with calcitriol. The study found that both groups achieved comparable results, with 45% of patients in each group reaching the target iPTH range of 150–600 pg/mL. Additionally, the hospitalization rates and survival outcomes were equivalent between the two groups (Thadhani, et al., 2020). Two studies indicated that doxercalciferol and paricalcitol were similarly effective in managing secondary hyperparathyroidism (SHPT) in hemodialysis patients. Zisman et al. found that doxercalciferol, when dosed at 55%–60% of the paricalcitol dose, achieved comparable suppression of iPTH levels, with similar profiles for serum calcium, phosphorus, and the incidence of hypercalcemia and hyperphosphatemia (Zisman, et al., 2005). Similarly, Fadem et al. demonstrated that converting patients from paricalcitol to doxercalciferol using a 65% dose conversion maintained iPTH levels within clinically satisfactory ranges and offered better phosphorus control, with a 28% reduction in treatment costs. Therefore, doxercalciferol is as effective as paricalcitol in controlling SHPT and may offer cost advantages (Fadem, et al., 2008). Our findings are consistent with the recent studies that have demonstrated the efficacy of doxercalciferol in reducing iPTH levels in dialysis patients with SHPT. However, the ACHIEVE trial (Fishbane et al., 2008) revealed that cinacalcet provided superior PTH control (68% vs. 52% reduction) compared to flexible vitamin D (doxercalciferol or paricalcitol), a finding supported by the larger EVOLVE trial (Chertow et al., 2012), which showed cinacalcet achieved 30% greater PTH suppression than vitamin D analogs. These results position doxercalciferol as equally effective to other vitamin D analogs but less potent than calcimimetics for severe SHPT.

Furthermore, the study showed that 56.82% of all patients achieved iPTH levels within the K/DOQI target range of 150–300 pg/mL, while only 33.33% of patients in the severe group reached this target. Hypercalcemia resulted in a reduction or temporary discontinuation of doxercalciferol in some patients, particularly those in the severe group, potentially leading to insufficient dosing and thus reducing the drug’s effectiveness in controlling iPTH levels.

### Impact on bone metabolism markers

In addition to iPTH, the study also assessed the impact of doxercalciferol on alkaline phosphatase (ALP), a marker of bone formation and turnover (Schini, et al., 2023).

After 12 weeks of treatment, ALP levels decreased in all three groups, with a statistically significant reduction observed in the severe group. This finding suggests that doxercalciferol may contribute to improved bone metabolism by reducing bone turnover, particularly in patients with more severe SHPT. Research by Parisi et al. (2003) has also highlighted the role of doxercalciferol in improving bone metabolism. In hemodialysis patients with secondary hyperparathyroidism, a 16-week treatment with doxercalciferol significantly decreased iPTH, bone-specific alkaline phosphatase, and serum type I collagen C telopeptide levels by 92%, 63%, and 53%, respectively. This treatment also increased bone mineral density (BMD) in the total skeleton, lumbar spine, and total femur by 6.5%, 6.9%, and 4.3%, respectively.

### Safety profile and adverse reactions

The safety profile of doxercalciferol was generally favorable, with an adverse reaction incidence rate of 37.8%. The most common adverse reaction was hypercalcemia, occurring in 33.3% of patients. However, only 8.9% of patients experienced severe hypercalcemia (serum calcium >2.8 mmol/L). Importantly, it was noted that after the administration of the study drug, the corrected serum calcium levels began to rise from the second week, with this increase persisting until the fourth week, after which they stabilized. When compared to the U.S. doxercalciferol trial (Tan, et al., 1997), the safety data from that trial reported a total of 8 cases (11.4%) of hypercalcemia, defined as serum calcium levels >2.8 mmol/L. However, it is important to note that the reference standard for hypercalcemia in this study was set at serum calcium levels >2.6 mmol/L, which is different from the original study. If we consider the incidence of serum calcium levels >2.8 mmol/L, this study had 4 cases (8.9%), which is slightly lower than that reported in the U.S. doxercalciferol trial. This is comparable to the incidence of hypercalcemia reported in other recent studies of vitamin D analogs in dialysis patients. Hypercalcemia is a common concern with vitamin D therapy (Järvelin and Järvelin, 2024). Literature reported that doxercalciferol showed a lower incidence of hypercalcemia and hypercalciuria than calcitriol (Gembillo, et al., 2021). The relatively low incidence of severe hypercalcemia is encouraging, as it suggests that doxercalciferol can be used safely with appropriate dose adjustments and monitoring.

Hyperphosphatemia was less common, affecting only 2.2% of patients in this study. During the study, the serum phosphorus levels of the subjects showed a fluctuating trend but basically remained stable, and there was no statistically significant difference in serum phosphorus levels before and after medication. This is an important finding, as hyperphosphatemia is a significant risk factor for cardiovascular disease in CKD patients (Ogata, et al., 2024). The study’s findings suggest that doxercalciferol may have a minimal impact on phosphorus levels, which is beneficial for patients with SHPT. Compared to the U.S. doxercalciferol trial (Tan, et al., 1997), the baseline serum phosphorus levels of the enrolled subjects in this study were essentially the same. After 12 weeks of treatment, the changes in serum phosphorus levels remained stable, consistent with the findings of the original study. This consistency further supports the conclusion that doxercalciferol has a minimal impact on phosphorus levels, which is advantageous for patients with SHPT as it reduces the risk of complications related to hyperphosphatemia.

The ability of doxercalciferol to effectively lower iPTH levels while maintaining a favorable safety profile is a significant advantage over traditional vitamin D analogs, which are often limited by their potential to induce hypercalcemia and hyperphosphatemia (Ketteler, et al., 2023).

### Clinical implications and future research

The results of this study have several clinical implications. Firstly, they support the use of intermittent intravenous doxercalciferol as an effective and relatively safe treatment option for SHPT in Chinese patients on MHD. The study provides evidence that doxercalciferol can effectively lower iPTH levels while maintaining a manageable risk of hypercalcemia. Secondly, the findings highlight the importance of individualized dosing and regular monitoring of calcium and phosphorus levels to optimize treatment outcomes and minimize adverse effects. Doxercalciferol demonstrates promising long-term benefits in SHPT treatment. It effectively suppresses parathyroid hormone while presenting a lower risk of hypercalcemia compared to calcitriol. For patients requiring sustained PTH control, doxercalciferol can reduce adverse effects on calcium-phosphate metabolism compared to calcitriol. Doxercalciferol is as effective as paricalcitol in controlling SHPT and its cost-effectiveness further supports its use in resource-limited settings (Fadem, et al., 2008). Emerging evidence suggests additional benefits, including positive effects on bone metabolism (Parisi et al., 2003) and a potential decrease in the risk of vascular calcification (Coburn et al., 2003). However, in view of the fact that all vitamin D analogues, including doxercalciferol, may induce hypercalcemia and hyperphosphatemia as adverse effects, for patients with SHPT who have concomitant hypercalcemia and/or hyperphosphatemia, it is recommended to utilize calcimimetics such as cinacalcet, either as monotherapy or in combination with vitamin D analogues (Fukagawa et al., 2013). Future research should focus on several areas. Long-term studies are needed to evaluate the sustained efficacy and safety of doxercalciferol in larger patient populations. Additionally, further research is warranted to explore the potential benefits of doxercalciferol on bone health and cardiovascular outcomes in CKD patients.

### Limitations

This study has several limitations that should be considered. Firstly, the sample size was relatively small, with only 45 patients enrolled and 44 completing the study, which may limit the generalizability of the findings to the broader population of Chinese maintenance hemodialysis (MHD) patients with secondary hyperparathyroidism (SHPT). Secondly, the study duration was short, lasting only 12 weeks, which is insufficient to assess the long-term efficacy and safety of intermittent intravenous doxercalciferol, including potential long-term side effects. Thirdly, the lack of a control group makes it difficult to definitively attribute the observed changes in iPTH and other parameters solely to doxercalciferol treatment. Furthermore, the assessment of bone health was limited to alkaline phosphatase levels, without more comprehensive measures such as bone mineral density or fracture incidence. Lastly, as the study was conducted in Chinese patients, the results may not be directly applicable to other ethnic groups or geographic regions due to potential differences in genetic, dietary, and environmental factors. Addressing these limitations in future research can provide a more comprehensive understanding of the role of doxercalciferol in managing SHPT in MHD patients.

## Conclusion

In conclusion, this study provides evidence that intermittent intravenous doxercalciferol is effective in reducing iPTH levels in Chinese MHD patients. In addition to lowering iPTH, doxercalciferol also contributed to improved bone metabolism by significantly reducing ALP levels in MHD patients with SHPT. The safety profile of doxercalciferol was generally favorable, with hypercalcemia being the most common adverse event. However, the incidence of severe hypercalcemia was relatively low, indicating that the treatment can be safely administered with appropriate dose adjustments and regular monitoring of calcium and phosphorus levels. This study is the first to evaluate the use of doxercalciferol in a Chinese population, addressing a significant gap in the literature and providing valuable insights into its potential as a therapeutic option for SHPT in Chinese MHD patients.

## Data Availability

The original contributions presented in the study are included in the article/supplementary material, further inquiries can be directed to the corresponding authors.
